# Recognition of Studied Words in Perceptual Disfluent Sans Forgetica Font

**DOI:** 10.3390/vision6030052

**Published:** 2022-08-24

**Authors:** Lucy Cui, Jereth Liu

**Affiliations:** 1 Department of Psychology, University of California, Los Angeles, CA 90095, USA; 2 Geffen Academy at University of California, Los Angeles, CA 90024, USA

**Keywords:** perceptual disfluency, disfluent font, memory

## Abstract

The new Sans Forgetica (SF) typeface creates perceptual disfluency by breaking up parts of letters vertically, horizontally, or diagonally, thereby fragmentizing them. While patterns of fragmentization are consistent for each unique letter, they are not uniform across letters. With Gestalt principles such as good continuation and perceptual completion being more difficult to implement in these settings, viewers may need to depend on context clues to identify words. This may be a desirable difficulty and improve memory for those words. Here, we investigate whether SF improves recognition of studied words. In Experiment 1, participants studied words in Arial and SF and completed old-new recognition tests where words retained their study fonts. In Experiment 2, we investigated the potential for context reinstatement—testing studied words in their studied fonts or the other font. Hit rate and discrimination sensitivities (*d’*) were analyzed for both experiments. Participants had significantly better recognition (hit rate) in SF than in Arial (Exp 1) and significantly higher discrimination sensitivities (*d’*) when words were tested in SF than in Arial (Exp 2). However, further examination of these results (e.g., marginally more response bias with SF than with Arial in Exp 1) lead us to hold reservations for the benefit of SF on word memory and conjecture that SF, at best, plays a limited role in improving recognition of studied words.

## 1. Introduction

Desirable difficulty refers to situations during learning when encoding of information is deliberately made difficult, but the encoded information becomes better retained and retrieved later [1]. Disfluent font, if shown to be a desirable difficulty, would be an easily adaptable and ready educational solution (e.g., [2,3]). With disfluent font, perceptual disfluency is manipulated through changing the characteristics of the font used, such as clarity (blurry words: [4]), color saturation (e.g., 15% and 25% grey scale: [5]), size (e.g., 5 point font: [6]), and typeface (e.g., [1]), or making fluent fonts harder to read (e.g., inverted words: [7]) or harder to process (e.g., perceptual interference using backward-masked words [8]).

Due to mixed findings across studies, whether perceptual disfluency can (reliably) improve educational outcomes is still debated. Recent meta-analyses are not encouraging. Xie et al. [9] gathered 25 empirical articles (totaling 3135 participants), that measured recall and had a fluent font comparison group, and found no effect of perceptual disfluency on recall (*d* = −0.01). Some researchers (e.g., [6,10]) attribute these null effects on a mismatch between an encoding process evoked during learning and a retrieval process required for the test and stress the importance of transfer-appropriate processing. Others (e.g., [3]) attribute the differences in results to the type of experimental manipulation—those that evoke increased top-down, higher-level processing (e.g., inverted words: [7]) find significant results but not low-level processing (e.g., blurry words: [4]). Blurring may cause readers to focus too much on the visual aspects of the stimuli, preventing them from engaging in deeper processing to encode orthography, phonology, and semantics [11].

### 1.1. What Makes SF Special

Typefaces used in previous studies include: 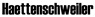 ([2] Experiment 2; grey scale: [12,13]), 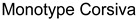 ([2] Experiment 2; [14] Experiment 1; [5] Experiment 2), 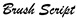 ([15] Experiment 1), 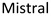 [16], and 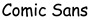 (grey scale: [17]). See Weisserber and Reinhard [10] for a detailed table of manipulations and effects from the recent literature. 

**Figure 1:**
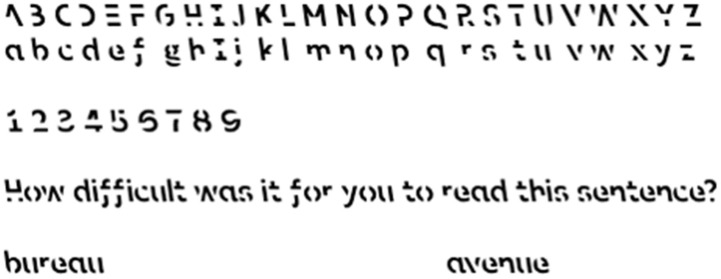
Sample of Sans Forgetica (SF) typeface. (SF is licensed under the Creative Commons Attribution-NonCommercial License, CC BY-NC; https://creativecommons.org/licenses/by-nc/3.0/ (accessed on 15 November 2018)).

SF was specifically designed with the concept of desirable difficulty in mind. The design team at RMIT University created this typeface, characterized by fragmented letters and digits (see Figure 1 for examples), to produce an optimal level of perceptual disfluency [18]. One of the researchers explained that SF works because people have an automatic tendency to complete the broken font and this “slows down the process of reading inside your brain. And then it can actually trigger memory” [19].

**Figure 2:**
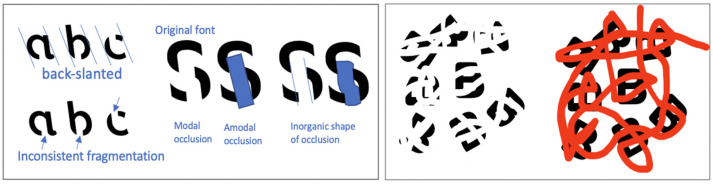
**Left** panel showing key characteristics of SF that creates visual difficulties. For example, the gap at letter c is positioned such that little continuation is suggested from the left to the right piece. Likewise, the gaps at letter S are positioned such that no strong connection is suggested by the three pieces. **Right** panel is a recreation of stimuli from Bregman [20].

Due to its specific visual/perceptual characteristics, Sans Forgetica (SF; see Figure 1) serves as an innovative test of disfluent font as a desirable difficulty in a way that previously studied typefaces (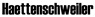, 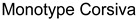, 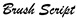, 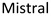) cannot. These other typefaces create perceptual disfluency through being an unfamiliar reading font or making individual letters hard to parse out (either through narrow spacing or conjoined lettering as in cursive typefaces). In addition, SF uses fragmented letters, where the same slashes of omission are used for the same letter but there is no regular pattern across letters. Moreover, SF’s letters are back-slanted while letters are front-slanted in italicizations and in most disfluent typefaces (See Figure 2, left panel). 

From the perspective of visual perception, these other typefaces produce a full signal for unfamiliar instances of known objects, whereas SF produces an impoverished signal for (unfamiliar instances of) known objects. Due to this impoverished signal, readers would need to rely on their perceptual systems to fill in the “gaps”. However, due to how SF is designed, readers cannot easily use perceptual grouping to recognize a letter. According to the Gestalt law of good continuation, if a straight line has a gap in the middle, the line is still perceived as a single line, but “good continuation” only works if the straight line is vertical or horizontal. If the line is oblique/slanted, then the two pieces of the line are perceived to be parallel with each other instead, namely, the Poggendorff illusion [21]. Since SF uses back-slanted letters, vertical strokes in letters are tilted and the perceptual continuation weakened. The gaps, sharing the same color as the background, make perceptual completion more difficult. When an object is occluding another object, we do not perceive the object underneath as being two separate objects. We perceive the two “fragmented” pieces to be one object and are able to perceive the missing contours of the object underneath [22]. Let us say we have a black capital letter B that is partially occluded by a red bar on a white background. The B can still be easily recognized, but if that red bar was changed to white to match the background, the resulting visual being how SF appears, completion becomes more difficult ([20]; See Figure 2, right panel). Without the perceived occlusion, completion becomes impossible. Recognizing the letter B may then rely on context clues, which engages higher-level processing. See Figure 2 (left panel) for characteristics of SF that create visual difficulties. In comparison to the manipulations of perceptual disfluency in the previous literature, it can be argued that SF utilizes perceptual degradation, perceptual distortion, and perceptual interference. Additionally, due to its novelty and impracticality of public use (e.g., presentations, posters), many participants would not have encountered the font before, maximizing the produced perceptual disfluency.

### 1.2. Existing Research on SF

To our knowledge, there have only been thirteen studies that investigated whether SF can be a desirable difficulty. These studies compare performance between SF and a fluent font, typically Arial, sometimes Times New Roman (e.g., [23]). SF seems to benefit recognition (greater sensitivity *d’)* of studied words ([24] Experiment 1—only when a test was not expected) but there are also mixed findings [25] and null results [26]. There are mixed results for recall of studied words ([27]—no benefit on free-recall of studied words from flashcards; [24] Experiment 2—benefit to cued-recall when test was not expected). Most studies use familiar words. One study [28] found that with learning new words, there was an SF effect for high-skill spellers but not for low-skill spellers. There are mixed findings as to whether SF improves recall of word-pairs: worse performance ([29] Experiment 3—lower judgment of learning; [30] highly-associated word-pairs using 500 ms presentation) and equivalent performance ([31] weakly-associated word-pairs). 

For longer text forms, the research findings have been more consistent. SF did not benefit memory for read information from passages ([30] Experiment 3—multiple-choice questions; [23] short-answer questions), recall of missing words in read passages over highlighted text ([31] Experiment 2), comprehension of passages ([30] Experiment 4, [32,33]), or solution rates of Cognitive Reflection Test problems [34]. However, there is one identified case where SF can be detrimental—proofreading text for errors. Cushing & Bodner [35] compared silent reading, reading out loud, and reading in SF font for the detection of contextual errors (i.e., grammar, word choice) and non-contextual errors (i.e., typos). They found that SF impaired the detection of non-contextual errors and did not help detection of contextual errors. 

### 1.3. Present Study

In the present study, we investigated whether SF benefits the recognition of studied words. The present study contributes to existing literature on SF by (1) using different methodological details and analyses than the previously published word recognition studies and (2) examining context reinstatement of the font used when studying by varying the fonts used during studying and testing. Arial was used as a control font because of its common usage in everyday life and in studies from this research area. 

## 2. Experiment 1: Memory for Words Using Transfer-Appropriate Processing

To assess improved memory, recognition was chosen over word recall for two main reasons: (1) the experimental set up would be analogous to vision experiments and allows for the interpretation of discrimination sensitivity and (2) some disfluency effects are found in recognition but not recall. It has even been proposed that recall tests are simply not sensitive to beneficial effects of small font size [6]. In addition, many manipulations of disfluent font are done through perceptual degradation (e.g., blurry words: [4], 10% grey-toned text: [14]) and perceptual interference (e.g., backward-masked words: [8]), which benefit recognition more than recall [4,36,37,38]).

The transfer-appropriate (TAP) framework has been used to explain when difficulties are desirable [39] and why some studies have not found a disfluency effect—the type of processing used during learning is incompatible with the type of retrieval required at test [6,10]. We applied the principle of TAP to maximize our chances of detecting an effect. Since we are simply presenting words to participants (with no additional task), we tested their memory by presenting those words and administering an old-new recognition test, where participants identify whether presented words were “old” (seen before) or “new” (not seen before). 

SF may elicit deeper processing of words at study and greater consideration of “oldness” during the testing phase, thereby increasing performance on a recognition test, compared to the automatic processing of words presented in legible and familiar font. The fragmented letters in SF may make word recognition more effortful, either by increasing the amount of processing at the letter level or increasing the number of attempts until the word is read correctly. The increased attention to letters (e.g., identifying (ambiguous) letters one-by-one) and the activation of more vocabulary in order to correctly identify the word may create additional memory traces for the studied word that can be used later on the recognition test. The more effortful (and slow) reading of words in SF may translate into more careful consideration of “oldness” during the testing stage since reading words is less automatic than with familiar, legible fonts. 

Alternatively, SF may hurt performance on a recognition test—the brief presentation may not be enough time for word recognition or extraction of enough features for regenerating words. Consequently, participants may need to remember the visual image of the word, which would be less reliable than remembering the word itself. 

In this experiment, we presented words briefly (1 s) in either SF or Arial during the study phase and administered an old-new recognition test where studied words retained their fonts from the study phase. Participants completed three blocks of study and test phases and each study phase consisted of 20 words. Our experiment is unique in its brief, fixed presentation time (vs. self-paced study trials of previous studies), shorter word set, and multiple blocks. Beyond examining the typical dependent measures of hit rate (true positive rate) and discrimination sensitivity, we also analyzed response bias and performance from block to block. 

### 2.1. Method

The study was conducted in accordance with the Declaration of Helsinki and approved by the Institutional Review Board of University of California, Los Angeles (protocol code 13-000078 and date of approval: 8 October 2017).

**Participants.** Sixty-six undergraduates from the University of California, Los Angeles (UCLA) were recruited from the psychology department’s subject pool and were compensated with partial course credit (either to fulfill a course requirement or for extra credit, depending on the course). There were no eligibility criteria for this study. 

Because there were no publications on SF when we were collecting data (data were collected in person prior to COVID-19), we used the upper bound of participants in a similar experimental design ([40], *n* = 54) as a reference for sample size for Experiment 1 and 2. Data collection went faster than expected so our sample sizes ended up being higher. The same recruitment method and participant compensation were used for Experiment 2. 

**Design.** This experiment had a within-subjects design, with font (Arial vs. Sans Forgetica) as the only independent variable.

**Materials.** The stimuli for this experiment consisted of 120 words generated from the English Lexicon Project database ([41] http://elexicon.wustl.edu (accessed on 15 November 2018)). While the chosen words could be nouns, adjectives, and/or verbs depending on context (e.g., “green” is a noun and adjective, “work” is a noun and verb), they all have noun as their primary part of speech. Words were 4 to 6 letters long and had 1 to 3 syllables. Their appearance frequency was between 42 and 50 per million words. Brief presentations of words have been shown to improve recognition due to participants’ need to fill in details of what they may have missed during the brief presentation, also known as perceptual interference effect. Because we considered SF to be more perceptual degradation than small font size, we had set stimuli presentation time to be a little longer than Halamish [6], which used 0.5 s.

**Figure 3:**
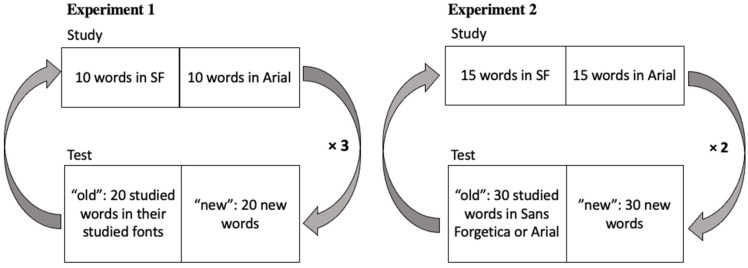
Experimental timeline for Experiment 1 (**left**; 3 blocks) and Experiment 2 (**right**; 2 blocks).

**Procedure.** Participants were told to remember the presented words. The experiment consisted of three blocks. In each block, participants studied 10 words in SF and 10 words in Arial, presented in random order for 1 s each, and then completed an old-new recognition task that consisted of 40 words: the 20 studied words in their studied fonts plus 10 new words presented in SF and 10 new words in Arial, presented one at a time (see Figure 3 for reference). Participants had unlimited time to identify the test word as “having seen it before” (“old”) or “not having seen it” (“new”). Participants completed the next block with a new set of study words and a new set of test words, with their fonts assigned randomly but evenly in the sets and their fonts counterbalanced across participants.

### 2.2. Results

Performance in Experiment 1 was assessed via hit rate, or proportion of studied words correctly recognized as “old” or studied; and the discrimination sensitivity (*d’)*, which takes into account false positive rates. A paired-samples *t*-test on *d’s* revealed no difference between SF condition (*M* = 2.32, *SD* = 0.66) and Arial (*M* = 2.26, *SD* = 0.77), *t*(65) = 0.71, *p* = 0.48. 

**Figure 4:**
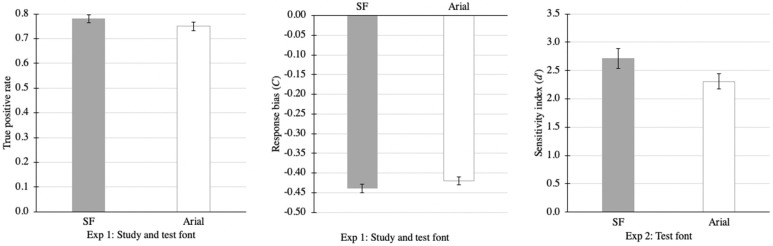
Summary of significant results from Experiment 1. Error bars represent standard error.

A paired-samples t-test on hit rates revealed a just-barely significant difference between SF and Arial, *t*(65) = 2.01, *p* = 0.048, Cohen’s *d* = 0.23. Participants had a slightly higher hit rate with SF (*M* = 0.78, *SD* = 0.12) than with Arial (*M =* 0.75, *SD* = 0.14). A significant difference in hit rates but no difference in *d’s* suggests that there may be a (greater) response bias in one condition over the other. We assessed response bias by computing the decision criterion: *C* = −[Z(true positive rate) + Z(false positive rate)]/2, where Z represents Z transformation. A negative *C* value means that the participant had a liberal decision criterion (responding more often “old”) whereas a positive *C* value means a conservative decision criterion (responding less often “old”), with zero representing no bias. A paired-samples t-test on *C* values revealed a marginally significant difference between the two font conditions, *t*(65) = 1.94, *p* = 0.057, Cohen’s *d* = 0.23. Participants had a more liberal criterion with SF (*M* = −0.44, *SD* = 0.09) than with Arial (*M* = −0.42, *SD* = 0.08). This analysis suggests that participants could have had a higher hit rate for SF because they were likely more biased toward identifying test words as “old” or “studied”. See Figure 4.

Though only marginally significant, participants’ stronger bias to identify words printed in SF as “old” could be a result of either misremembering a studied word (or having a false memory of another word) or being uncertain whether the word was studied but leaning enough toward “old” to select it. It is possible that more words were activated in the working memory during the studying of words printed in SF (either words that were lexically or semantically similar or words that the participant “regenerated” in attempts to read the presented word) and these activated words formed a false memory of having studied said words. Participants may have had a more liberal criterion for SF because of the perceived disfluency—participants believe their memory for studied words is weaker and compensate (for anticipated weaker performance) by saying a word was studied when unsure. Whatever the reason may be, SF seems unideal in situations where false positives matter, like standardized tests that have guessing penalties—losing points for wrong answers instead of just not receiving points, but debatably better in situations where only true positives matter. 

**Block-by-block.** Given that participants were not pre-exposed to SF prior to the experiment, we wanted to check whether there were any differences in behavior (hit rate, *d*’, bias) with SF between the first block and the subsequent blocks (averaged). While there was a significant difference in hit rate, *t*(65) = 5.11, *p* < 0.001, and *d’*, *t*(65) = 4.69, *p* < 0.001, between the first block and subsequent blocks for SF, the same was true for Arial (hint: *t*(65) = 3.31, *p* = 0.002, *d*’: *t*(65) = 3.90, *p* < 0.001). To better understand what is going on, we ran a 2 (font: SF vs. Arial) × 3 (blocks) repeated-measures ANOVA on hit rate, *d*’, and bias. All ANOVAs passed Mauchly’s Test of Sphericity. Regarding hit rate, there was a marginally significant main effect of font, *F*(1, 130) = 3.96, *p* = 0.05, η_p_^2^ = 0.06, observed power = 0.50), a significant main effect of block (*F*(2, 130) = 26.83, *p* < 0.001, η_p_^2^ = 0.29, observed power = 1.00), where hit rate decreased significantly from block to block, *p* < 0.001, and no interaction, *F*(2, 130) = 0.27, *p* = 0.77. It is not surprising that hit rates decreased from block to block, as worsening performance is expected as more word sets are studied, likely due to proactive interference.

A closer look at the effect of font for each block reveals that the hit rate difference was only marginally significant in the first block, *t*(65) = 1.82, *p* = 0.07, while the second and third blocks were not significant, *t*(65) < 0.92, *p* > 0.36. Regarding *d’*, there was a significant main effect of block (*F*(2, 130) = 11.10, *p* < 0.001, η_p_^2^ = 0.15, observed power = 0.99), where *d’* decreased significantly from block to block, *p* < 0.001, but no main effect of font, *F*(130) = 0.11, *p* = 0.74, or interaction, *F*(2, 130) = 0.11, *p* = 0.90. Regarding bias, there was no main effect of font, *F*(1, 130) = 1.66, *p* = 0.20, or block, *F*(2, 130) = 1.72, *p* = 0.18, and no interaction, *F*(2, 130) = 0.04, *p* = 0.96. This block-by-block analysis draws doubt on the significant difference in hit rate we found on the experiment as a whole (i.e., all blocks combined). Since only the first block of trials had a marginally significant difference and the remaining two blocks did not have a reliable difference, this suggests that the overall effect was mostly driven by the difference in the first block and averaging with the remaining two blocks just so happened to make the font difference more reliable.

The marginally significant difference in the first block and no reliable difference in the remaining two blocks could be interpreted as due to the moderating effect of test expectancy found in Geller and Peterson [24]. In that study, the high test expectancy was set by the instructions: “your memory will be tested for words in different typefaces” while low test expectancy was set by instructions: “you will be reading words in different typefaces”. In our study, we asked participants to remember the words that are presented to them without an explicit mention of a test, which could be considered medium test expectancy, as asking someone to remember something suggests that they could be tested on it later but not necessarily (directly). However, after the first block, test expectancy could have been solidified in the participants. Thus, this provides an explanation for why there was a marginally significant difference in font for the first block but not the remaining two blocks (i.e., lower test expectancy in the first block than the remaining ones).

With this being said, we would like to caution that this interpretation is not the only one possible. Given that the hit rate difference only occurred in the first block, and possibly due to response bias, it could also be due to a learning effect. Namely, participants readjusted their decision criterion after the first block so that no hit rate difference between the two fonts remained in the subsequent blocks. 

## 3. Experiment 2: Memory for Words When Varying Study and Test Fonts, Potential for Contextual Reinstatement?

In Experiment 1, we tried to maximize the chance of finding an effect of font by using transfer-appropriate processing principles. Considering that SF was developed to be a study tool for students and it is not expected that exams will be printed in SF, we were interested in the situation where study font and test font do not match, since it is likely that a student would study in SF but be tested in a fluent font like Arial. In Experiment 2, we investigated whether SF could benefit encoding and/or retrieval by varying the font used in the study phase (encoding) and the font used in the test phase (retrieval). A natural curiosity from this experimental setup is the potential of contextual reinstatement—does the font (e.g., SF) provide a contextual cue for recognizing a studied word as “old”? We assessed for this possibility by analyzing the results via match-mismatch conditions.

The desirable difficulty argument for disfluent font emphasizes deeper and more elaborate processing, or the encoding stage. Typically, a disfluent font is manipulated during the encoding stage and a legible font is used for testing. We expected studying in SF to lead to better performance on a recognition test regardless of test font. We were not anticipating test font to make a significant difference in recognition test performance because we did not have reason to believe that the font of the test words would differentially help participants retrieve studied words. 

Opposing the potential of contextual reinstatement (i.e., better recognition when the study font and test font match) is another possibility—an advantage of testing in a different font than the font used to study. We compared match (when study font and test font match) and mismatch conditions. While the principle of transfer-appropriate processing may suggest that match conditions would outperform mismatch conditions, the change in font in mismatch conditions may help participants make old-new decisions during the recognition test—seeing words studied in Arial but tested in SF may promote more careful consideration of “oldness” and seeing words studied in SF but tested in Arial may alleviate some working memory demands of reading SF. 

### 3.1. Method

**Participants.** Fifty-eight fresh UCLA undergraduates participated in-person since this was done prior to COVID-19. 

**Design.** Experiment 2 had a 2 (study font: Arial vs. SF) × 2 (test font: Arial vs. SF) within-subjects design. 

**Materials.** Words used here were from the same database as Experiment 1. The number of trials per block was changed from Experiment 1 to maintain the total number of studied words at 60. Experiment 1 had three blocks of 20 studied words (60 total) and Experiment 1 had two blocks of 30 studied words (60 total). Two blocks in Experiment 2 were used for counterbalance purposes.

**Procedure**. Participants were told to remember the presented words. The experiment consisted of two blocks: (1) study in the two fonts (SF, Arial) and test in SF, and (2) study in two fonts and test in Arial, the order of which was counterbalanced. For each block, participants first studied 30 words, 15 of which were randomly assigned to be displayed in SF and the other 15 in Arial. (The font assignment was also randomized from one participant to the next.) Each word was presented for 1 s and one at a time. After the study phase of each block, participants completed an old-new recognition task. This test consisted of 60 words: 30 studied words and 30 new words, all displayed in one font (SF or Arial), see Figure 3 for a reference. The test items were presented one at a time, in a random order, with no time limit for responses. Participants completed the next block with a new set of words, with the test font different than that of the first block. 

### 3.2. Results

Performance was similarly assessed via hit rate, and the discrimination sensitivity (*d’*).

**Correct identifications.** We conducted a 2 (study fonts) × 2 (test fonts) repeated-measures ANOVA on participants’ hit rates (true positive rates). Participants made numerically more correct recognitions of words studied in Arial (*M* = 0.79, *SD* = 0.13) than words studied in SF (*M =* 0.77, *SD* = 0.13), but this difference was not significant, *F*(1, 57) = 1.22, *p* = 0.27. Whether test words were displayed in SF (*M* = 0.78, *SD* = 0.12) or Arial (*M* = 0.78, *SD* = 0.14) made no difference, *F*(1, 57) = 0.001, *p* = 0.97. There was also no interaction, *F*(1, 57) = 0.84, *p* = 0.36.

**Discrimination sensitivity***d’* was calculated separately for the two independent variables: study font and test font. *d’* of study font would be Z(Hit of words studied in Arial from both blocks)−Z(False Alarm of words studied in Arial from both blocks). A concern for the *d’* calculation is false positive rates of zero. Among the 58 total participants, 15 had zero false positives when the test font was SF. Six of the 15 also had zero false positives when the test font was Arial. We used a typical correction method for zero false positives [42] and adjusted the false positive rate to be 1/(2*n*), where *n* = 30 was the number of signal trials in that block. After these corrections, we calculated *d’*s for each study font and each test font. 

While a paired-samples *t*-test of *d’s* for study font showed no difference (SF: *M* = 2.23, *SD* = 0.88, Arial: *M* = 2.29, *SD* = 0.91), *t*(54) = 1.28, *p* = 0.21; a paired-samples t-test of *d’s* for test font revealed a significant difference, *t*(57) = 2.42, *p* = 0.02. Participants had significantly higher *d’* when studied words were tested in SF (*M* = 2.71, *SD* = 1.35) than when studied words were tested in Arial (*M* = 2.31, *SD* = 1.04; see Figure 4). Degrees of freedom differ because three participants had infinity as *d’s* for study font—a result of (close to) perfect true positive rates and close to zero (after correction) false positive rates. However, the statistical significance persisted even after these three participants were excluded, *t*(54) = 2.21, *p* = 0.032. Nevertheless, the effect size with and without the three participants removed from analysis was weak (Cohen’s *d* = 0.32–0.33).

Finding a main effect of test font but not study font suggests that SF does not necessarily strengthen the encoding of studied words but possibly supports successful retrieval. This result further supports that SF changes the nature of one’s consideration of whether tested words were previously studied, much like in Experiment 1. No difference in hit rates and a greater *d’* for SF as a test font suggests that bias was no longer an issue in Experiment 2. The variation of test font with respect to study font may have led participants to be more conservative in their judgments of whether a tested word was previously studied, perhaps because the font of the tested words could no longer serve as a cue for recognition.

**Response Bias.** We assessed response bias by computing the decision criterion: *C* = −[Z(true positive rate) + Z(false positive rate)]/2 and conducted a 2 (study font: Arial vs. Sans Forgetica) × 2 (test font: Arial vs. Sans Forgetica) repeated-measures ANOVA on response bias. Greater values of *C* represent more conservative decision criteria (responding less often “old”). There was a nonsignificant difference between study fonts Arial (*M* = 0.34, *SD* = 0.46) and Sans Forgetica (*M* = 0.37, *SD* = 0.50), *F*(1, 57) = 1.19, *p* = 0.28, and a nonsignificant interaction, *F*(1, 57) = 1.56, *p* = 0.22. However, there was a significant difference between test fonts Arial (*M* = 0.27, *SD* = 0.44) and Sans Forgetica (*M* = 0.44, *SD* = 0.60), *F*(1, 57) = 6.47, *p* = 0.014, η_p_^2^ = 0.10, observed power = 0.71, with participants being more conservative on words tested in Sans Forgetica. There were no order effects, *F*(1, 56) = 0.29, *p* = 0.59.

**Match-Mismatch comparisons.** We collapsed conditions to form match (study and test font match) and mismatch groups. There was no difference between match conditions (*M* = 0.79, *SD* = 0.16) and mismatch conditions (*M* = 0.77, *SD* = 0.15) on hit rate, *t*(115) = 0.89, *p* = 0.37, or on *d’* (match: *M* = 2.72, *SD* = 1.48; mismatch: *M* = 2.57, *SD* = 1.36; *t*(115) = 1.49, *p* = 0.14), suggesting no contextual reinstatement. We also made comparisons within match conditions (i.e., study Arial test Arial vs. study SF test SF) and within mismatch conditions. *d’* for studying in Arial and testing in SF (*M* = 2.79, *SD* = 1.52) was higher than for studying in SF and testing in Arial (*M* = 2.36, *SD* = 1.15), *t*(57) = 2.09, *p* = 0.04. This difference supports our interpretation of the main effect of test font—SF supports careful consideration of old-new. All other comparisons for hit rate and *d’* were not significant, *t*(57)s < 0.94, *p*s > 0.35. 

## 4. General Discussion

SF serves as an innovative test of disfluent font because it creates perceptual disfluency of a different nature than previous manipulations—one that encourages higher-level processing and introduces inconsistent perceptual difficulty (i.e., fragmentizing) as opposed to lower-level manipulations (e.g., blurring). While existing studies in this area create perceptual disfluency by lowering the quality of text (e.g., low contrast, small font) or by using hard-to-read typeface (e.g., narrow spacing or conjoined letters as with cursive), SF creates perceptual disfluency by fragmentizing letters in an irregular way (i.e., pieces that are missing are not the same across letters) and back-slanting those letters. These characteristics make it difficult for viewers to use Gestalt principles such as good continuation and perceptual completion to identify letters. Reading words, in turn, may depend more on context clues when letters are ambiguous individually. 

Given the novelty of SF, there are limited number of existing studies. Of those studies, there are mixed results on whether SF can benefit memory for studied information (e.g., words, phrases, passages). We contribute to this growing literature by (1) using different methodological details (i.e., fixed presentation time, shorter word sets, and multiple blocks) and analyses (e.g., response time, block-by-block analyses) than the previously published word recognition studies and (2) examining context reinstatement of the font used during studying by varying the fonts used during studying and testing. We expected SF to encourage higher-level lexical processing thereby improving recognition of studied words when the tested words retained their studied fonts (Exp 1) and when the font of the tested words varied (Exp 2).

Initial examination of the results suggests that SF improves the recognition of studied words but further examination suggests this interpretation is premature and oversimplified. In Experiment 1, when the studied words retained their font in testing, participants did have significantly higher hit rates for SF than for Arial (consistent with findings from Geller et al. [28] Experiment 3 though we collected our data prior to its preprint/publication), but an examination of response bias suggests that better hit rate cannot be interpreted as better memory. While the difference in response bias was only marginally significant, participants seemed slightly more biased to identify test words as “old” when they were printed in SF, which explains the higher hit rate. The greater response bias may suggest some uncertainty or overcompensation for it. 

However, this response bias did not occur in Experiment 2. While there was marginally more bias to respond “old” with words studied and tested in SF than in Arial in Experiment 1, in Experiment 2 there was significantly less bias to respond “old” with words tested in SF than in Arial, regardless of what font they were studied in. The additional variation of font at test may have encouraged participants to be more conservative or careful about their decisions of “old” or “new”. In addition, less response bias was coupled with higher sensitivity, meaning participants were not only less biased but also more accurate in general. 

These results suggest that SF may affect how studied words were perceptually encoded and subsequently retrieved, but it does not necessarily mean that SF assists in the retrieval process or improves word memory. This interpretation is consistent with Hu et al. [26], which using a drift diffusion model (DDM) to split response time of a recognition test into a decision component related to retrieval and a non-decision component unrelated to retrieval, found that SF had a greater non-decision time and that a greater non-decision time was related to lower confidence ratings. Therefore, SF may only change participants’ consideration of whether a word was previously studied and not the retrieval process. This consideration may be affected by whether study and test font were the same (Experiment 1) or whether study and test font varied (Experiment 2, additional layer of difficulty) because the different testing conditions may influence one’s confidence and one’s subsequent responses (biased or conservative).

Our block-by-block analysis of Experiment 1 also limits any strong interpretation of its results. When performance across the three blocks was averaged, there was a significant difference in hit rate but when blocks were analyzed individually, only the first block had a difference with marginal significance. One possible explanation for this is that there was less test expectancy in the first block than the remaining two blocks and test expectancy has been identified as a moderator of the SF effect on studied words [24]. Experiment 2 had some surprising results. We had expected only a main effect of study font, such that words studied in SF would be recognized better than those studied in Arial, regardless of test font. This prediction is based on the belief that SF would influence encoding and not retrieval. Instead, we found a main effect of test font—participants had significantly higher sensitivity when they were tested using SF than using Arial, regardless of study font. This result suggests that SF aids in the accurate consideration of a word’s “oldness”, or word recognition. The effect size, however, was small/weak. Although it is always possible that the study is underpowered; given our *n* = 58 and our Cohen’s *d* of 0.32–0.33, we conjecture that an effect of SF, if it exists, cannot be bigger than what our data show. This caps the effect of SF on memory and hence shows SF’s limitation. 

We had also expected contextual reinstatement (or encoding specificity)—testing in a studied font would be better than testing in a different font, with the alternative hypothesis being that variation may encourage more careful consideration of “oldness” or reduce working memory demands of SF, thereby improving recognition performance. We did not find evidence for either hypothesis. Instead, the only significant difference found in our match-mismatch analyses was that sensitivity was significantly higher when studying in Arial but testing in SF (*M* = 2.79, *SD* = 1.52) than studying in SF but testing in Arial (*M* = 2.36, *SD* = 1.15), but similar to studying and testing in SF (*M =* 2.83, *SD* = 1.48). This suggests that SF is just better as a test font. 

Why did SF not generate as substantial benefit in word memory as anticipated? We speculate that, at essence, SF is letter based, whereas word memory is based on words. As a result, possibly, the difficulty working out the identities of the letters is not directly related to word memory, since the spatial sequences or patterns of letters that form into words are beyond the visual perceptual disfluency of individual letters. This may in part be why prior studies manipulating the ease of letter visibility, as opposed to letter sequences, produced contradictory results in regards to whether their perceptual disfluency facilitated memory or not. There is indeed evidence that words with 20% scrambled letters benefited recognition [10]. Our reasoning could also explain the consistent null results found with reading passages ([23,30] Experiment 3, [31,32,33])—SF only creates letter-level difficulty, once the word(s) are identified/read, SF does not interfere or support the memory for the ideas those words conveyed. This letter-level difficulty could also be why SF made non-contextual errors (i.e., typos) in passages from Cushing & Bodner [35] more difficult to detect. There may be confusability between similar looking SF-letters that made typos more difficult to detect, but this impairment could also enlighten us on how people read words in SF. Reading words in SF may depend on context clues and not require individual letter identification, which is worth exploring further.

Our analyses lead us to hold reservations for the benefit of SF on word memory and conjecture that SF, at best, plays a limited role in improving word recognition. However, research on SF as a potential desirability difficulty is far from conclusive and whether SF can create a perceptual disfluency effect remains controversial. Much research is still needed, but researchers should proceed with caution and be very deliberate about where and how to pour resources into this area of research. Given the novelty of SF, it is understandable to want to compare it to a fluent font in every typical education-related task, but it may be more informative to understand how SF compares to other (disfluent) fonts in how it is processed. Future studies may benefit from better understanding of how people process SF. This will help us understand or explain the existence of an effect or lack thereof and better anticipate which domains SF may benefit. Eskenazi and Nix [28] was a good starting point—through eye-tracking data, they found that participants skipped significantly more words when reading text printed in Courier (fluent font) than text printed in SF and that total reading time for SF text was significantly longer only for participants with high spelling skills. Their data counter the common assumption that all people read SF slower than a fluent font. 

SF was developed to be a study tool, so to protect students from fruitless efforts in incorporating SF into their studying, it is still worthwhile to identify domains where SF could be used as a study tool and caution against those where SF may not be helpful. Research in this area may benefit from out-of-the-box thinking—not simply asking in which domains SF is beneficial but how SF can be used in those domains. One good example of this is Cushing and Bodner [35] who investigated whether SF can be used to detect errors when proofreading one’s written drafts. Given our results of better word recognition when tested in SF, perhaps an application can be using the font to test oneself after one has already studied the material in a fluent font, almost like using SF as a cued recall. However, this possibility would need to be investigated further and on more ecologically valid material (i.e., stimuli closer to what students would be studying for classes) than ours. The possibilities could be endless and we should not limit ourselves to what has been done with other disfluent typefaces. 

## Data Availability

The data presented in this study are openly available in OSF at 10.17605/OSF.IO/7PFY5.
